# The Imperative for Patient-Centered Clinical Decision Support

**DOI:** 10.5334/egems.259

**Published:** 2018-05-30

**Authors:** Laura Haak Marcial, Joshua E. Richardson, Beth Lasater, Blackford Middleton, Jerome A. Osheroff, Kensaku Kawamoto, Jessica S. Ancker, Danny van Leeuwen, Edwin A. Lomotan, Shafa Al-Showk, Barry H. Blumenfeld

**Affiliations:** 1 RTI International, US; 2 Apervita, US; 3 TMIT Consulting, US; 4 University of Utah, US; 5 Weill Cornell Medical College, US; 6 Health Hats, US; 7 Agency for Healthcare Research and Quality, US

**Keywords:** clinical decision support (CDS), patient-centered clinical decision support (PCCDS), learning network, patient-centered clinical decision support learning network (PCCDS Learning Network)

## Abstract

This commentary introduces the Patient-Centered Clinical Decision Support (PCCDS) Learning Network, which is collaborating with AcademyHealth to publish “Better Decisions Together” as part of *eGEMs*. Patient-centered clinical decision support (CDS) is an important vehicle to address broad issues in the U.S. health care system regarding quality and safety while also achieving better outcomes and better patient and provider satisfaction. Defined as CDS that supports individual patients and their care givers and/or care teams in health-related decisions and actions, PCCDS is an important step forward in advancing endeavors to move patient-centered care forward. The PCCDS Learning Network has developed a framework, referred to as the Analytic Framework for Action (AFA), to organize thinking and activities around PCCDS. A wide array of activities the PCCDS Learning Network is engaging in to inform and connect stakeholders is discussed.

## Introduction

This commentary introduces the Patient-Centered Clinical Decision Support (PCCDS) Learning Network, which is collaborating with AcademyHealth to publish “Better Decisions Together” as part of *eGEMs*. Patient-centered clinical decision support (CDS) is an important vehicle to address broad issues in the U.S. health care system regarding quality and safety while also achieving better outcomes AND better patient and provider satisfaction. We define PCCDS as CDS that supports *individual* patients and their care givers and/or care teams in health-related decisions and actions. The PCCDS Learning Network has developed a framework, referred to as the Analytic Framework for Action (AFA) to organize thinking and activities around PCCDS. The PCCDS Learning Network offers a wide array of activities to inform and connect stakeholders and advance PCCDS-related endeavors to move patient-centered care forward.

Despite significant advances in medical science over the past five decades, the quality of medical care in the United States continues to leave significant room for improvement. For example [1], in their influential 2003 study, McGlynn et al. studied over 17,000 patients and determined that just under 55 percent of patients received recommended care. Another important aspect of health care quality is its safety, and there are many different approaches to measuring this [2]. One thing is certain, regardless of the actual numbers few industries would allow for the death rates that are ascribed to medical errors in the United States even at the lowest estimate [3]. While deaths due to medical errors are different from the delivery of recommended care, the two are linked. Clinical decision support can help assure that recommended care is delivered, while decreasing the potential for medical errors. Moreover, the value of health care (defined as the health outcomes achieved relative to costs) [4] also has significant room for improvement, especially in the United States, as compared to many other peer nations.

Given this recognized need for improved care quality, safety, and value, there is no stakeholder with more at stake than the patient. Empowering patients to control aspects of their care goes a long way to improving care quality. Research findings support this: compared with patients that do not, patients who participate in medical decisions tend to report more satisfaction with their care [5], enjoy a better quality of life [6], and are adherent to recommendations [7] with greater satisfaction [8].

Research in clinical decision support (CDS), defined as processes for enhancing health-related decisions and actions [9] using evidence-based information [10], has shown that the use of CDS can reduce costs, improve quality, and reduce medical errors in clinical settings [11,12]. Optimally, CDS provides patient-specific information and knowledge, enabled by health information technology (IT), to clinicians, patients, or other individuals to enhance health and health care [13]. CDS interventions draw from a rich palette of options that ideally make the right thing to do easy [9]. To date, these systems have focused on the delivery of information to clinicians more than patients [14].

CDS interventions improve care processes and outcomes when they achieve the CDS Five Rights—i.e., deliver the right information to the right people through the right formats via the right channels at the right times [15]. CDS has not reached its full potential in driving care transformation in part because opportunities to support *patients* (as a ‘right person’ for information delivery) haven’t been fully explored and cultivated [16]. Another problem is that because CDS has traditionally been delivered to the clinician, patient-related factors have not been incorporated. In addition, researchers developing the evidence and guidelines from which CDS is derived do not always sufficiently take into account non-medical determinants of health such as social determinants, patient-specific variables (e.g., patient history factors), comorbidities, genetic information and patient preferences and values. These factors are shown to impact patient and provider decision-making in the context of preference-sensitive decisions like prostate care for men [17,18]. CDS often isn’t clear about the population the evidence was drawn from, making it challenging to apply CDS guidance to individual patients (who may or may not be representative of the population).

Examples of PCCDS include highlighting care needs (such as for disease screening and management) in individuals and populations through dashboards and registry reports that incorporate patient preference information and provide tailoring options for patient and provider to customize. This would be complemented by addressing those needs through evidence-based order sets and judicious use of notifications tied to appropriate actions. When well implemented, CDS can improve health care processes and outcomes [12]. These efforts serve to help drive progress toward the quadruple aim: to enhance the patient experience, to improve population health, to reduce costs, and to improve the work life of health care providers [19].

## PCCDS: The Next Logical Phase in the Advance of CDS

As noted above, PCCDS is CDS that supports *individual* patients and their care givers and/or care teams in health-related decisions and actions, and it does this by leveraging information from patient-centered outcomes research (PCOR) findings and/or patient-specific information (e.g., patient-generated health data). Establishing this definition and source criteria has helped set in motion a critical shift from provider-centric to patient-centric CDS.

To assure alignment with the patient perspective, we have asked patient advocates to join the PCCDS Learning Network. Identifying what works for patients, caregivers, and clinicians (people at the center of care) as they make medical decisions together in the context of their life flow and workflow is a critical component. We will strive to engage people at the center of care in conversations about real-life, pragmatic opportunities to bridge population-based evidence with individual circumstances. One measure of success will be patient and clinician advocacy organizations feeding and referring opportunities to develop or implement PCCDS to the PCCDS Learning network as a nexus for disseminating information, providing input, and impacting how CDS can actively support patient-centered care.

For example, evidence shows that patients with a family history of breast cancer due to the BRCA gene, value genetic counseling on the need for genetic testing, the interpretation of results, and a dialog with clinicians about treatment pathways and outcomes data [20,21]. Since almost 60 percent of women with the harmful PALB2 mutation will develop breast cancer by age 70, patients highly value discussing the risks and prevention strategies at the right time with clinicians [22]. Because this exercise in shared decision making can occur at crucial times in the lives of affected women, an approach involving evidence on patient outcomes is especially important [23]. In a modern shared decision-making scenario, both patient and provider share critical diagnostic and treatment information bidirectionally and engage in a partnership that elicits clinical options and patient preferences, and facilitates an informed decision by the patient [24]. This might include a co-created care plan (analogous to an asthma care plan) which outlines evidence-based treatment choices and decisions discussed and decided upon jointly.

In another example, PCCDS intervention for blood pressure monitoring could support patients gathering home blood pressure readings and transmitting the readings to their care team. For example, the PCCDS intervention can remind patients of specific actions to take based on the reading, as dictated by the care plan developed with their provider. The PCCDS can be used to notify the provider about any out-of-range value(s) and provide support for further action and discussion by the patient and provider about the readings and associated treatment(s). In this way, the PCCDS intervention can facilitate patient-led self-management as well [25]. For hypertension, medication adherence is crucial and “patient empowerment can promote medication adherence, but it requires a co-constructed sense of control in the doctor-patient dyad” [26].

Similarly, for a patient living with chronic pain who is on pain management drugs daily, information about non-pharmacologic alternatives, daily dosing and non-opioid medications, and other avenues for pain relief may be useful. A smartphone application, shared between patient and provider, could reinforce safe morphine dosing information, provide pain level self-monitoring tools, and link to other pain management resources [27]. Together, these activities would serve to reinforce behavior change and support the patient and provider in ongoing efforts to optimize the pain control care planning and results.

## A Learning Network to Advocate for PCCDS

Our society now expects information at our fingertips to help make informed decisions. Indeed, just as we expect that we can easily identify the best value for airline travel options or our current bank balance via intelligent devices and information systems, we expect no less from our health care system [28]. Together, these activities would serve to reinforce behavior change and support the patient and provider in ongoing efforts to optimize care planning and results.

The PCCDS Learning Network was formed to accelerate the dissemination of patient-centered research findings through CDS. The PCCDS Learning Network highlights innovative ways to implement evidence into knowledge artifacts and services that promote patient-specific decisions, fundamentally turning evidence into action. Supported by a four-year, Agency for Healthcare Research and Quality (AHRQ) funded cooperative agreement, the purpose of the PCCDS Learning Network is to inform and connect PCCDS stakeholders, and to thereby broadly advance PCCDS initiatives and their value in making care more patient centered and achieving the Quadruple Aim. These stakeholders include a variety of entities including (and not limited to):

- patients and their caregivers,
- patient advocates,
- clinicians and care teams,
- standards development organizations,
- CDS developers and implementers,
- health IT and CDS vendors,
- payers and regulators, and
- subject matter experts and researchers.

For example, the PCCDS Learning Network engages CDS Connect, which is an AHRQ initiative to make CDS more shareable, standards-based, and publicly available. It includes a national repository of CDS, a CDS authoring tool, application programming interfaces, and implementation guidance to help make CDS development and implementation more systematic and replicable. In its first year, the CDS Connect project developed CDS, using the HL7 Clinical Quality Language, that was implemented as a shared-decision making tool for clinicians and patients to discuss cholesterol management. The PCCDS Learning Network’s goal is to advance activities like CDS Connect—e.g., by helping ensure evidence-based tools made available within that initiative are used broadly and effectively by those they can benefit, while providing input to help ensure that the artifacts provided meet stakeholder needs.

The PCCDS Learning Network has an open call for participants in the network and initially successfully enrolled scores of active volunteer members in both leadership and membership activities of the organization. Since its inception, the PCCDS Learning Network has operationalized a working definition for PCCDS, produced a webinar series to disseminate PCCDS efforts and research, held an annual conference on PCCDS, and developed an online collaboration environment for PCCDS stakeholders to engage on important topics in the area. In addition, an environmental scan conducted in the first year of funding helped establish a baseline for the extant literature in this area.

**Figure 1:**
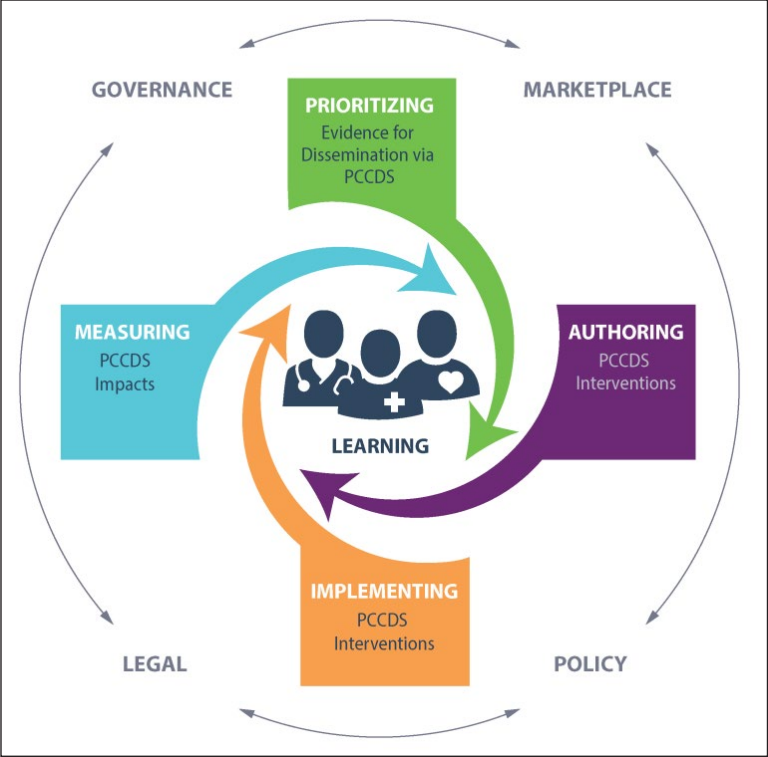
Analytic Framework for Action Depicts Interacting Components for Disseminating Patient-centered Evidence via PCCDS.

The scan also led the development of an approach to organize efforts, the Analytical Framework for Action (AFA). The AFA depicts a lifecycle of interacting components for developing and disseminating evidence-based research findings via PCCDS (See Figure 1). Key factors of the AFA include:

- PRIORITIZING: Applying objective measures of evidence for identifying and prioritizing findings that are to be transformed and disseminated via PCCDS, assessing or defining their implementability, and defining stewardship and governance requirements.
- AUTHORING: Applying accepted data and knowledge standards for translating findings into one or more PCCDS intervention types that support key decisions, actions, and communications that are essential to ensuring that the finding improves care and outcomes.
- IMPLEMENTING: Applying standardized, best practice methods and architectures for operationalizing PCCDS interventions into clinical workflows that deliver the right information to the right people in the right formats through the right channels at the right times to improve care processes and outcomes (“CDS Five Rights”) [15].
- MEASURING: Ensuring that PCCDS interventions measurably improve clinician and patient decision-making, care processes, and outcomes.
- LEARNING: Aggregating local PCCDS-related outcomes and effectiveness measures to facilitate both local and system-level learning from identified gaps in PCOR knowledge, and lessons learned from authoring, implementing, and using PCCDS in clinical practice to enhance care and outcomes.
- EXTERNAL FACTORS: External factors including the marketplace, policy, legal, and governance issues that impact development, dissemination, and implementation processes for PCCDS.

We use the AFA to orient our discussions with patients, clinicians, policymakers, and others to determine where important work has already been done, where there are gaps in the development of PCCDS activities, and where there are opportunities to do more.

The PCCDS Learning Network explored self-monitoring of blood pressure (SMBP) as an initial working group use case in its first year. Findings from that working group include and understanding that adaptation of guidelines for use as CDS can be complicated. It was hard to find SMBP guidelines as a single source, in machine readable format, or with machine readable logic, and the process of converting them is a difficult and sometimes impossible task. Delivering the guidelines to the patient adds yet another layer of complexity. This highlighted the need to ascertain the fit for a given guideline as an implementable form of PCCDS. The AFA provides a framework to assist in organizing and prioritizing guidelines that are well suited to PCCDS.

As a result, towards the end of its second year, the PCCDS Learning Network used the AFA as the organizing model for the PCCDS Learning Network Resource Center which disseminates information about PCCDS resources and initiatives. The Resource Center is a growing library of links within key categories that provide access to resources such as PCOR findings and evidence-based guidelines to enhance clinical care that could be implemented via PCCDS; and rules, methods, or utilities that could be used for structuring or delivering PCCDS interventions. Anyone can contribute to the PCCDS Learning Network Resource Center via our website: https://pccds-ln.org.

## Enhancing the Value from PCCDS in 2018

The PCCDS Learning Network is engaging stakeholders by promoting PCCDS solutions in high profile clinical domains such as the opioid epidemic and in chronic disease. The opioid crisis is currently devastating communities throughout the United States. Among the efforts toward combating the crisis are initiatives aimed at developing CDS interventions to help clinicians, patients, and their caregivers promote the appropriate use of opioid analgesics appropriately [29]. Recognizing the impact of this crisis, in 2018 the PCCDS Learning Network will be focusing on one overarching objective: leading the development of a National Action Plan (NAP) for PCCDS to help address the opioid crisis. The goal is to delineate a framework that lays out a connected set of actions and steps necessary to make high quality PCCDS available in high priority use cases, and facilitate the collaboration between stakeholders needed to make such use a reality. The key deliverable will be a published manuscript proposing a coordinated set of actions to be taken to ameliorate the opioid crisis. Another important result from the NAP development process will be connections and dialog that seed NAP execution. The NAP, while tailored for the opioid crisis, will also serve as a template for bringing similar PCCDS initiatives forward within specific domains.

### Working Groups to Explore Challenge Areas

The PCCDS Learning Network is chartering three working groups in 2018 to advance knowledge and action in specific areas fundamental to delivering value from PCCDS broadly, and to the development and execution of the NAP in particular:

1. A trust framework working group aimed at addressing trust barriers and facilitators as they pertain to the exchange of clinical knowledge and its use for PCCDS. This working group will primarily address the external factors as outlined in the AFA, the marketplace, and the policy, legal, and governance issues that impact development, dissemination, and implementation of clinical knowledge artifacts. For example, this working group will address questions like:
  - Do I trust this author of the CDS knowledge artifact I’m interested in?
  - Do I trust that it is encoded and stored correctly in the CDS Connect repository?
  - Do I trust that it is implemented correctly in my EHR, or via a cloud service to my EHR?
  - Do I trust that I can monitor the impact or efficacy of this CDS artifact in my practice?
  The findings will specifically inform the CDS Connect project’s governance and design decisions and will provide a framework for sharing the clinical knowledge artifacts within the repository. This working group will also outline a broad trust framework for developing and using clinical knowledge in PCCDS.
2. An opioid NAP working group aimed at achieving consensus around a highly desirable future vision for PCCDS-enabled pain management and opioid use, and defining and building momentum toward stakeholder actions to achieve this vision. This working group will consider all key AFA factors to ensure that the recommended approach to PCCDS-enabled pain/opioid management addresses pertinent improvement drivers. Key deliverables for this working group include the development of an NAP document that outlines and begins driving action – via key engaged stakeholders – toward broader and more effective PCCDS implementation to improve pain management and opioid use.
3. A technical framework working group that will address factors related to the technical challenges of developing and implementing PCCDS. This working group will focus on the Authoring and Implementing key factors of the AFA and will detail the technical challenges of authoring and implementing PCCDS (using the opioid medication management use case) and provide a working proof of concept demonstration of a PCCDS implementation to address the opioid crisis.

### Annual Conference

The Annual Conference (to be held in Washington, D.C., in Fall 2018) will focus on the progress made towards the PCCDS Learning Network’s year 3 goals and bring together experts and interested stakeholders to share their efforts to highlight, cultivate, and disseminate PCCDS. The conference will also serve as an important forum to communicate and refine the NAP.

### Education and Dissemination

Continuing our bimonthly webinar series, the focus of the PCCDS Learning Network’s webinars in 2018 will be on patient-centered approaches to the development of CDS and on the opioid NAP and chronic disease.

The PCCDS Learning Network has also launched a special section, “Better Decisions Together,” in *eGEMs*, an online and open-access journal that features peer-reviewed articles in decision support and electronic health data. “Better Decisions Together” was established to share, showcase, and create a dialog around innovative research that advances the science of PCCDS. The special section will disseminate compelling PCCDS findings, methods, and strategies to shrink the gap between research and use in patient care. The first call for papers ended in February 2018, yet new submissions are continuously accepted on a rolling basis. This first edition of the new section is an important forum for the PCCDS Learning Network in establishing a research agenda around PCCDS. “Better Decisions Together” was established to share, showcase, and create a dialog around innovative research that advances the science of PCCDS.

## Conclusion

The work of the PCCDS Learning Network in 2018 will focus on highlighting efforts that leverage PCCDS for opioid medication management and in the prevention of chronic diseases. Development of the NAP, a framework for PCCDS to address the domestic opioid crisis, will be integrated throughout our yearlong activities with representation in the working groups, in the webinars, and at our annual conference. The goal is to sufficiently seed PCCDS with an opioid focus, so that moving similar efforts into other domains is relatively straightforward. Publications will be primary work products resulting from the development of the NAP and the efforts of the working groups and will be disseminated at conferences and in peer-reviewed journals. “Better Decisions Together” will be leveraged to report on barriers, relevant research, and solutions for the PCCDS community going forward.

The mission of the PCCDS Learning Network, is to create an ecosystem that allows all stakeholders to reduce the friction of turning evidence based research into patient centered, CDS-enabled actions that produce better care and outcomes. This requires input and engagement from our growing community. Our experiences over the last two years have led us to a focus on patient-centered CDS that also enhances provider and care giver experiences. In 2018, the PCCDS Learning Network will focus its efforts to highlight, cultivate, and disseminate PCCDS that addresses the opioid crisis and promotes evidence-based ways to empower patients and their care teams, to make better decisions together.

**Getting Involved with the Learning Network to Advance PCCDS—Join Us**

You can get involved by becoming a member on ourwebsite, submittinga resource to our Resource Center, submitting a manuscript to “Better Decisions Together,” attending our webinars, and coming to our annual conference. With your help, the PCCDS Learning Network will continue to broadly facilitate the dissemination and use of patient-centered, evidence-based research through PCCDS. We welcome your involvement and your input.
